# Interleukin-8 (IL-8) as a Potential Mediator of an Association between Trimethylamine N-Oxide (TMAO) and Proprotein Convertase Subtilisin/Kexin Type 9 (PCSK9) among African Americans at Risk of Cardiovascular Disease

**DOI:** 10.3390/metabo12121196

**Published:** 2022-11-30

**Authors:** Alyssa M. Baginski, Nicole Farmer, Yvonne Baumer, Gwenyth R. Wallen, Tiffany M. Powell-Wiley

**Affiliations:** 1 Translational, Biobehavioral, and Health Disparities Branch, National Institutes of Health Clinical Center, Bethesda, MD 20892, USA; 2 Social Determinants of Obesity and Cardiovascular Risk Laboratory, Division of Intramural Research, National Heart, Lung, and Blood Institute, Bethesda, MD 20892, USA; 3 Intramural Research Program, National Institute on Minority Health and Health Disparities, Bethesda, MD 20892, USA

**Keywords:** cardiovascular disease, TMAO, PCSK9, inflammation

## Abstract

Trimethylamine N-oxide (TMAO)—a microbial metabolite derived from the hepatic–gut axis—is linked to inflammation, hyperlipidemia, and cardiovascular disease (CVD). Proprotein convertase subtilisin/kexin type 9 (PCSK9), which is largely hepatically expressed, blocks low-density lipoprotein (LDL) receptor recycling, also leading to hyperlipidemia. The primary objective of this study was to investigate a previously hypothesized potential relationship between TMAO and PCSK9 in order to explore novel mechanisms linking TMAO and CVD risk. African American adults at risk of CVD living in the Washington DC area were recruited to participate in a cross-sectional community-based study (*n* = 60, 93% female, BMI = 33). Fasting levels of inflammatory cytokines (i.e., interleukin (IL)-1 beta, tumor necrosis factor-alpha, and interleukin-8), TMAO, and PCSK9 were measured using Luminex and ELISA, respectively. Univariate and multivariate linear regression analyses and structural equation mediation analyses were conducted using STATA. All models were adjusted for body mass index (BMI) and atherosclerotic CVD risk score (ASCVD). A significant association between TMAO and PCSK9 was identified (β = 0.31, *p* = 0.02). Both TMAO and PCSK9 were significantly associated with IL-8 (TMAO: β = 0.45, *p* = 0.00; PCSK9: β = 0.23, *p* = 0.05) in adjusted models. Mediation analysis indicated that 34.77% of the relationship between TMAO and PCSK9 was explained by IL-8. Our findings indicate a potential PCSK9-involved pathway for TMAO and CVD risk, with potential mediation by IL-8.

## 1. Introduction

**Figure 1:**
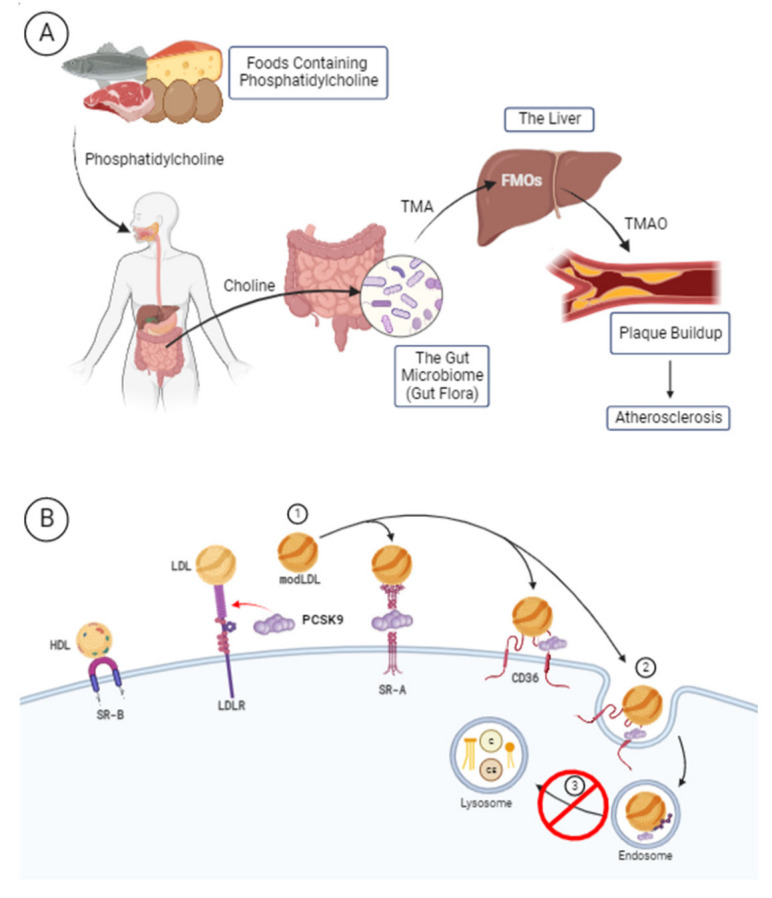
(**A**) Endogenous production of TMAO from dietary precursors. (**B**) Mechanism of action of PCSK9 in the disruption of LDL receptor recycling. Created with BioRender.com (accessed on 9 November 2022).

Trimethylamine N-oxide (TMAO) is a microbial metabolite that serves as a molecular chaperone, stabilizes proteins to regulate hydrostatic pressure, influences protein conformational changes, participates in allosteric regulation, and modulates intracellular molecular effects [1,2,3,4]. These functions take place largely in the endothelium of the liver, intestines, and arterial walls [2]. In vitro murine model studies, using peritoneal macrophages, have shown that treatment with TMAO results in an increase in the release of inflammatory cytokines (e.g., IL-1β, TNFα) and atherogenesis, partially due to enhanced accumulation of cholesterol in macrophages and subsequent foam cell formation [5,6]. TMAO is found exogenously (preformed) within marine life. Endogenous production also occurs and involves the hepatic–gut axis in response to dietary consumption of precursor nutrients, such as eggs and red meats containing phosphatidylcholine (Figure 1A) [7]. For example, in the gut, choline is cleaved from larger molecules and taken up by bacteria residing in the gut microbiome, such as *Clostridium* XIVa strains and *Eubacterium* sp. strain AB3007 of the Firmicutes phylum [8]. These microbes then produce trimethylamine (TMA), which is transformed into TMAO by hepatic flavin-containing dimethylaniline monooxygenases (FMOs)—specifically FMO3—in the liver. Preformed TMAO can also be obtained in the diet from fish consumption. Elevated levels of TMAO—especially endogenously formed TMAO—have been linked to inflammation and cardiovascular disease (CVD) in both murine and human models [7,9]. Particularly relevant is the link between TMAO and the CVD risk factor hyperlipidemia (i.e., elevated blood cholesterol levels) through alterations in reverse cholesterol transport or enhanced macrophage foam cell formation [10]. Other mechanisms linking TMAO to hyperlipidemia have not been reported to date.

Proprotein convertase subtilisin/kexin type 9 (PCSK9) is a largely hepatically expressed protein that regulates blood cholesterol levels [11]. The mechanism of action through which PCSK9 maintains or alters cholesterol levels is by binding low-density lipoprotein (LDL) and preventing both LDL and LDL receptor recycling from taking place (Figure 1B). A recent study adds to these findings by demonstrating that PCSK9, when incorporated into the HDL particle, enhances LDLR binding and potentially worsens its impact on hyperlipidemia [12]. Overexpression of PCSK9 in atherosclerotic murine models showed that the PCSK9-mediated pro-atherosclerotic impact is LDLR-dependent, highlighting the importance of PCSK9 in LDLR expression and subsequent hyperlipidemia [13]. In addition to its impact on LDLR and circulating lipid levels, PCSK9 has been directly linked to inflammation and pro-atherosclerotic processes, as it was demonstrated to be produced by various cell types crucial to atherogenesis (e.g., endothelial and smooth muscle cells), regulating apoptosis, and stimulation of pro-inflammatory cytokines and foam cell formation [14].

TMAO and PCSK9 have previously been connected to one another as the result of investigation into gut dysbiosis and atherosclerotic cardiovascular disease [15]. Presently, due to their connections to the hepatic environment and hyperlipidemia, respectively, the primary objective of this study was to investigate a potential relationship between TMAO and PCSK9 in order to explore a novel mechanism linking TMAO and CVD risk. We hypothesized that TMAO and PCSK9 would have a direct association among a sample population of African American adults at increased risk of CVD, and that this relationship would be mediated by inflammatory cytokines.

## 2. Materials and Methods

African American adults at risk of CVD living in the Washington DC area were recruited to participate in a cross-sectional community-based study to evaluate cardiovascular health and the feasibility of digital-technology-enabled cardiovascular health behavior monitoring; a subset of the study population underwent clinical examination and collection of fasting blood samples, as described previously [16]. Relevant to the current analysis, serum samples were analyzed for clinical CVD risk factors. Fasting levels of inflammatory cytokines (i.e., interleukin (IL)-1 beta (β), tumor necrosis factor-alpha (TNFα), IL-8, IL-18, interferon gamma (IFNγ)) were measured using multiplex ELISAs (Meso Scale Diagnostics, Rockville, MD, USA), while TMAO and PCSK9 were measured using ELISAs from Biohippo (BHE12105704) and BioLegend (443107), respectively. Univariate and multivariate linear regression analyses and structural equation modeling analyses for assessing mediation were conducted using STATA. All models were adjusted for body mass index (BMI) and 10-year atherosclerotic CVD risk score (ASCVD) [17].

## 3. Results

**Figure 2:**
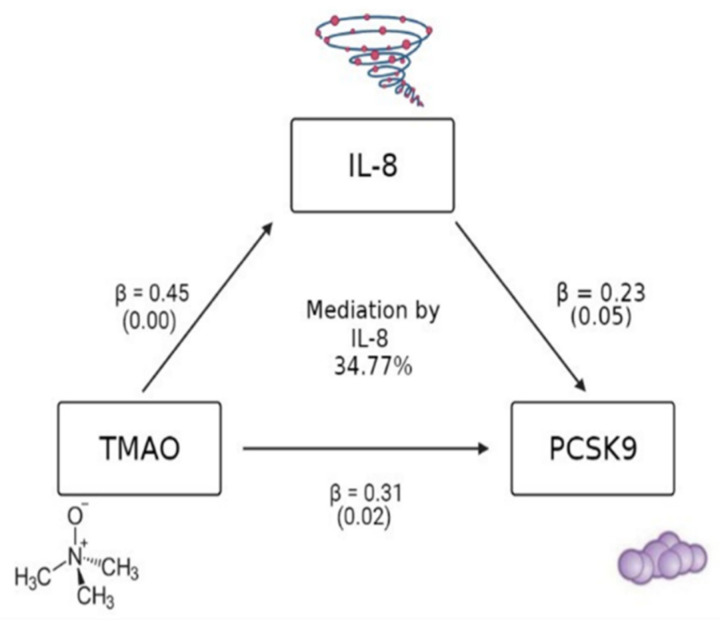
Model of the mediation of the observed relationship between TMAO and PCSK9 by IL-8. Model adjusted for BMI and ASCVD risk score, (*n* = 60). ASCVD: atherosclerotic cardiovascular disease; BMI: body mass index; IL: interleukin; TMAO: trimethylamine N-oxide; PCSK9: proprotein convertase subtilisin/kexin type 9. Created with BioRender.com (accessed on 25 February 2022).

The participant sample (*n* = 60) included self-identified African Americans who presented, on average, as having obesity (mean BMI = 33 ± 7.85 kg/m^2^), with a majority self-identifying as women (*n* = 56, 93.33%) (Table 1). Of the tested biomarker associations, a significant association between TMAO and PCSK9 was identified in both unadjusted (β = 0.30, *p* = 0.02) and adjusted (β = 0.31, *p* = 0.02) models. Of the cytokines measured, IFNγ (β = 0.39, *p* = 0.00) and IL-18 (β = −0.32, *p* = 0.03) were associated with PCSK9 in adjusted models, while IL-1β (β = 0.36, *p* = 0.01) and TNFα (β = 0.43, *p* = 0.00) were associated with TMAO in adjusted models (Supplementary Table S1). IL-8 was the only cytokine to be significantly associated with both TMAO and PCSK9 (TMAO: β = 0.43, *p* = 0.00 and β = 0.45, *p* = 0.00; PCSK9: β = 0.27, *p* = 0.04 and β = 0.23, *p* = 0.05) in both unadjusted and adjusted models (Supplementary Table S1). Mediation analysis indicated that 34.77% of the relationship between TMAO and PCSK9 was explained by IL-8 when TMAO was framed as the independent variable of the relationship (Figure 2). There was no statistically significant mediation between TMAO and PCSK9 by IL-8 when PCSK9 was framed as the independent variable (mediation = 0.3477 or 34.77%; *p* = 0.099).

## 4. Discussion

Identifying specific pathways by which TMAO confers CVD risk is important for developing therapeutic interventions that can mitigate the clinical effects of TMAO. Our findings indicate a potential pathway by which TMAO may promote hyperlipidemia—a CVD risk factor—through PCSK9. Furthermore, our results suggest that this pathway is mediated by the inflammatory cytokine IL-8, which is consistent with previously published studies by our group and others indicating a modulatory role of TMAO on inflammatory cytokines involved in CVD risk among adults [18,19].

The hypothesized pathway by which TMAO is positively associated with PCSK9 through modulation of IL-8 is intriguing, as TMAO and IL-8 are involved in the gut–hepatic axis [20] and, thus, could have direct effects on the largely hepatically expressed PCSK9. One potential mechanism by which this could occur, which requires further study with a larger sample and a longitudinal study design, is the triggering of nuclear factor-kappa B (NF-κB) and NLR family pyrin domain-containing 3 (NLRP3) inflammasomes by TMAO, which subsequently increases inflammation and cytokine production [21]. The above steps are illuminated by murine models, which suggest that pro-inflammatory cytokines have regulatory power over PCSK9, even as they themselves are regulated by it [22,23,24]. IL-8 is also related to atherosclerosis through its participation in and driving of inflammatory cascades [25] and macrophage differentiation [26], which may play a role in the relationship as well.

There may be other mechanisms connecting TMAO and PCSK9 beyond a mediation by IL-8, which are worth investigating. Another such mechanism involves the gut–heart axis and the impact of gut dysbiosis on the respective levels of these molecules and on CVD itself [15]. There are many specific types of bacteria that influence the onset of CVD—for example, a higher ratio of Firmicutes to Bacteroides or the reduction in butyrate-producing bacterial groups. Additionally, this altered microbiome can affect TMAO through an increase in Proteobacteria and other TMA producers, and PCSK9 through conditions such as small intestinal bacterial overgrowth (SIBO) or irritable bowel syndrome (IBS), which are both correlated with increased PCSK9 production. Interestingly, therein lies another opportunity for interaction between TMAO and PCSK9, as the SIBO and IBS increase PCSK9 production through the activation of toll-like receptor 4 (TLR4) induction which, incidentally, is also activated by TMAO [15,27].

In terms of potential interventions, levels of both TMAO and IL-8 can be reduced through dietary modifications, such as the consumption of a Mediterranean diet [2,28]. Additionally, reducing or eliminating foods that are high in TMAO, choline, carnitine, betaine, and ergothioneine—such as marine fish or red meats—can also result in a reduction in TMAO levels [2]. Given the connection of both TMAO and IL-8 to diet and inflammation, it may be reasonable to consider upstream targets, addressed through diet, to mitigate levels of PCSK9. Despite the fact that PCSK9 is now an available therapeutic pharmaceutical target [29], access to PCSK9 inhibitors is linked to one’s type of health insurance and, thus, may not be widely accessible [30]. Therefore, alternative methods to control PCSK9 levels may be advantageous.

Future studies may consider identifying the role of dietary factors that could decrease IL-8 and potentially influence the TMAO–PCKSK9 relationship. Additionally, investigation into the mechanisms by which these regulatory relationships occur should also take place. Long-term goals from these results include finding novel therapeutic targets for CVD and its associated comorbidities, potentially through dietary interventions.

## Figures and Tables

**Table 1:** Participants’ characteristics.

	Mean (SD)/Total *n* (%)
African American	60 (100)
Sex, Female	56 (93.33)
Age (years)	60.83 ± 10.52
Type 2 Diabetes Mellitus History	13 (21.67)
Hyperlipidemia History	33 (55.00)
Smoking History	7 (11.67)
BMI (kg/m^2^)	33.00 ± 7.85
LDL (mg/dL)	105.5 ± 33.02
HDL (mg/dL)	66.57 ± 20.58
Triglycerides (mg/dL)	84.97 ± 26.43
Total Cholesterol (mg/dL)	188.98 ± 35.20
TMAO (ng/mL)	37.54 ± 72.61
PCSK9 (ng/mL)	326.52 ± 83.66
ASCVD 10-Year Risk Score * (%)	10.75 ± 8.51
IL-8 (pg/mL)	4.54 (2.63–5.37) **
IFNγ (pg/mL)	8.83 (3.37–10.01) **
IL-18 (pg/mL)	399.998 (265.78–496.29)
IL-1β (pg/mL)	0.19 (0.10–0.22) **
TNFα (pg/mL)	1.61 (1.25–1.88) **

* ASCVD risk score calculated from sex, age, race, total cholesterol, HDL-C, systolic blood pressure, personal history of diabetes, personal history of smoking, and personal history of treatment for hypertension. LDL: low-density lipoprotein; HDL: high-density lipoprotein; ASCVD: atherosclerotic cardiovascular disease; IL: interleukin TNF: tumor necrosis factor; TMAO: trimethylamine N-oxide; PCSK9: proprotein convertase subtilisin/kexin type 9. ** Interquartile ranges.

## Data Availability

The data presented in this study may be available upon request and determination made by the corresponding and senior authors. Data are not publicly available due to restrictions on sharing data in accordance with the consent provided by the participants.

## Supplementary Materials

The following supporting information can be downloaded at: https://www.mdpi.com/article/10.3390/metabo12121196/s1, Table S1: Results of cytokine association analysis.
