# Supplementary material for: Interleukin-8 (IL-8) as a Potential Mediator of an Association between Trimethylamine N-Oxide (TMAO) and Proprotein Convertase Subtilisin/Kexin Type 9 (PCSK9) among African Americans at Risk of Cardiovascular Disease
Source: Metabolites. 2022 Nov 30;12(12):1196. doi: 10.3390/metabo12121196 (PMC9785610; doi:10.3390/metabo12121196)
Supplement: Supplementary file 1 [file metabolites-12-01196-s001.zip › metabolites-1996095-supplementary.pdf]

Variable	BioMarker	Unadjusted Beta-value	Unadjusted P-value	Adjusted[1] Beta-value	Adjusted[1] P-value
**TMAO**	PCSK9	0.2984	0.023*	0.3149	0.017*
**IL-8**	PCSK9	0.2780	0.035*	0.2581	0.051
**IL-8**	TMAO	0.4284	0.001**	0.4537	<0.001**
**IL1-β**	PCSK9	0.1015	0.452	0.9967	0.470
**IL1-β**	TMAO	0.3541	0.006**	0.3592	0.007*
**TNFα**	PCSK9	0.1014	0.449	0.0995	0.459
**TNFα**	TMAO	0.4442	0.000**	0.4284	0.001*
**IFNγ**	PCSK9	0.4150	0.001**	0.3893	0.002*
**IFNγ**	TMAO	0.0824	0.535	0.0895	0.495
**IL-18**	PCSK9	-0.3338	0.035*	-0.3247	0.034*
**IL-18**	TMAO	-0.0794	0.617	-0.0708	0.655

[1] Adjusted for ASCVD risk score and BMI.

Table S1: Results of Cytokine Association Analysis. ASCVD: atherosclerotic cardiovascular disease; BMI: body mass index; IL: interleukin; TNF: tumor necrosis factor; TMAO: Trimethylamine N-Oxide; PCSK9: Proprotein Convertase Subtilisin/Kexin Type 9. * $p<0.05$, ** $p<0.001$
